# Neuroprotective Effects of Herbal Ethanol Extracts from *Gynostemma pentaphyllum* in the 6-Hydroxydopamine-Lesioned Rat Model of Parkinson's Disease

**DOI:** 10.3390/molecules15042814

**Published:** 2010-04-16

**Authors:** Hyun Sook Choi, Mi Sook Park, Seung Hwan Kim, Bang Yeon Hwang, Chong Kil Lee, Myung Koo Lee

**Affiliations:** 1 College of Pharmacy and Research Center for Bioresource and Health, Chungbuk National University, Cheongju 361-763, Korea; E-Mails: 6151494@hanmail.net (H.S.C); parkms@cnuh.co.kr (M.S.P); byhwang@chngbuk.ac.kr (B.Y.H); cklee@chungbuk.ac.kr (C.K.L); 2 College of Physical Education, Kyunghee University, Youngin 449-701, Korea; E-Mail: barkleyk34@khu.ac.kr (S.H.K)

**Keywords:** *Gynostemma pentaphyllum*, 6-hydroxydopamine-lesioned rats, tyrosine hydroxylase, dopamine, Parkinson’s disease

## Abstract

6-Hydroxydopamine administration for 28 days (8 μg/2 μL) reduced the number of tyrosine hydroxylase (TH)-immunopositive neurons to 40.2% in the substantia nigra compared to the intact contralateral side. Dopamine, 3,4-dihydroxyphenylacetic acid, homovanillic acid and norepinephrine levels were reduced to 19.1%, 52.3%, 47.1% and 67.4% in the striatum of 6-hydroxydopamine-lesioned rats compared to the control group, respectively. However, an oral administration of herbal ethanol extracts from *Gynostemma pentaphyllum* (GP-EX) (10 mg/kg and 30 mg/kg) starting on day 3 post-lesion for 28 days markedly ameliorated the reduction of TH-immunopositive neurons induced by 6-hydroxydopamine-lesioned rat brain from 40.2% to 67.4% and 75.8% in the substantia nigra. GP-EX administration (10 and 30 mg/kg) also recovered the levels of dopamine, 3,4-dihydroxyphenylacetic acid, homovanillic acid and norepinephrine in post-lesion striatum to 64.1% and 65.0%, 77.9% and 89.7%, 82.6% and 90.2%, and 88.1% and 89.2% of the control group. GP-EX at the given doses did not produce any sign of toxicity such as weight loss, diarrhea and vomiting in rats during the 28 day treatment period and four gypenoside derivatives, gynosaponin TN-1, gynosaponin TN-2, gypenoside XLV and gypenoside LXXIV were identified from GP-EX. These results suggest that GP-EX might be helpful in the prevention of Parkinson’s disease.

## 1. Introduction

Parkinson’s disease (PD) is a commonly occurring age-related neurodegenerative disorder which is accompanied by the symptoms of muscular rigidity, bradykinesia, rest tremor and loss of postural balance [1]. The basic neuropathology of PD involves the degeneration of the dopaminergic nigrostriatal tracts with a corresponding decrease in the levels of dopamine and its metabolites 3,4-dihydroxyphenylacetic acid (DOPAC) and homovanillic acid (HVA), and norepinephrine [2].

In the dopamine biosynthetic pathway, 3,4-dihydroxyphenylalanine (L-DOPA) is formed from L-tyrosine by tyrosine hydroxylase (EC 1.14.16.2, TH), a rate-limiting enzyme. L-DOPA is then converted to dopamine by aromatic L-amino acid decarboxylase (EC 4.1.1.28). L-DOPA is, therefore, the most-prescribed therapy for controlling the symptoms of PD [1,3]. However, chronic prolonged therapy for PD with L-DOPA results in a loss of drug efficacy and irreversible adverse effects and subsequently leads to the development of motor complications such as fluctuation and dyskinesia [4]. Such adverse effects limit the current symptomatic therapy for PD and have fueled the search for new PD treatments with non-dopaminergic alternatives or substances that will relieve the L-DOPA-induced adverse actions for PD. 

6-Hydroxydopamine (6-OHDA), a specific dopaminergic neurotoxin, has been commonly used to produce experimental animal models of PD [5]. The stereotaxic injection of 6-OHDA into the substantia nigra, medial forebrain bundle and striatum of the brain injures dopaminergic neurons selectively through the formation of various reactive oxygen species (ROS), lipid peroxidations, damaged proteins, and amino acid modifications [6,7,8]. 6-OHDA leads to a reduction in glutathione content and superoxide dismutase and catalase activities in the striatum [9]. In addition, it is reported that L-DOPA treatment results in endogenous 6-OHDA formation in rat brain [10]. 

*Gynostemma pentaphyllum* (Cucurbitaceae) is usually used as an herbal tea and is widely believed to have various protective and/or improving functions for diabetes, depression, anxiety, fatigue, hyperlipidemia, immunity, oxidative stress and tumors [11]. The major constituents of *G. pentaphyllum* have been isolated and reported as many gypenosides [11]*.* GP-EX has been also found to have an anti-stress function and immunomodulatory activity in mice [12,13]. However, the *in vivo* functions of *G. pentaphyllum* for medical applications have not been elucidated precisely, especially related to antioxidant and immune stimulation.

The present study was, therefore, designed to investigate the beneficial effects of *G. pentaphyllum* in a rat model of 6-OHDA-lesioned PD. The dopaminergic neuronal cell death induced by 6-OHDA lesion in rats was blocked by co-treatment with the ethanol extracts from the leaves of *G. pentaphyllum* (GP-EX), which was examined by histochemical (TH-immunoreactive cells) and neurochemical (dopamine, DOPAC, HVA and norepinephrine levels) techniques. 

## 2. Results

### 2.1. TH-immunopositive neuronal cell survival in 6-OHDA-lesioned rats by GP-EX administration

Rats administered 6-OHDA for 14–28 days showed the symptoms of PD, which was confirmed by an excess of 150–300 contralateral rotations in a 60 min period after treatment with apomorphine (0.5 mg/kg) in the 6-OHDA-lesioned rats.

**Figure 1:**
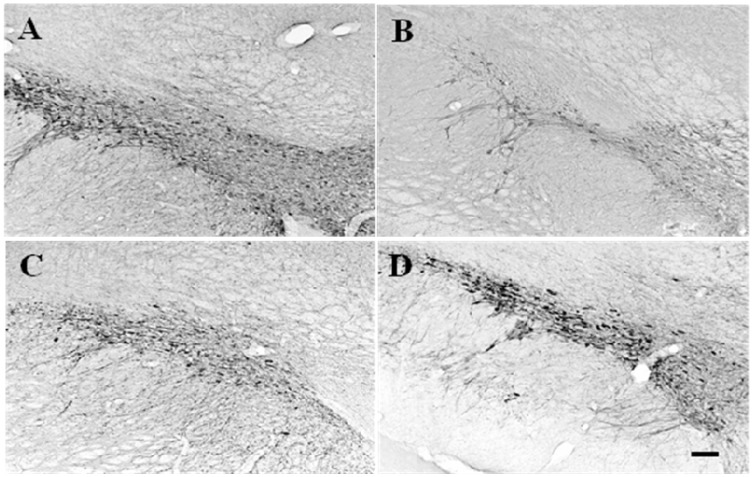
Photomicrographs of TH immunoreactivity on substantia nigra tissue sections from representative rats of each group. Those that received 6-OHDA lesions (B) exhibited a significant loss of dopaminergic neurons compared to sham-lesioned rats (A) while 10 mg/kg GP-EX administration for 28 days (C) and 30 mg/kg GP-EX administration for 28 days (D) in 6-OHDA-lesioned rats showed less loss of dopaminergic neurons. Scale bar = 100 μm.

TH-immunopositive neurons within the substantia nigra of rats injected with saline did not display any reductions compared to normal areas and the cells were either poly- or ovoid-shaped (Figure 1A). TH-immunopositive neurons in the left substantia nigra were markedly reduced in rats administered 6-OHDA for 28 days (Figure 1B). Substantia nigra near the 6-OHDA-lesioned areas displayed drastic reductions in TH-immunopositive neurons compared to control groups. The TH-cells in the substantia nigra on the 6-OHDA-lesioned side were small with thinner processes and uneven color. However, co-administration with GP-EX at 10 and 30 mg/kg (p.o.) from day 3 post-lesion for 28 days ameliorated the loss of TH-immunopositive neurons by 6-OHDA lesions in substantia nigra (Figure 1C and D).

**Figure 2:**
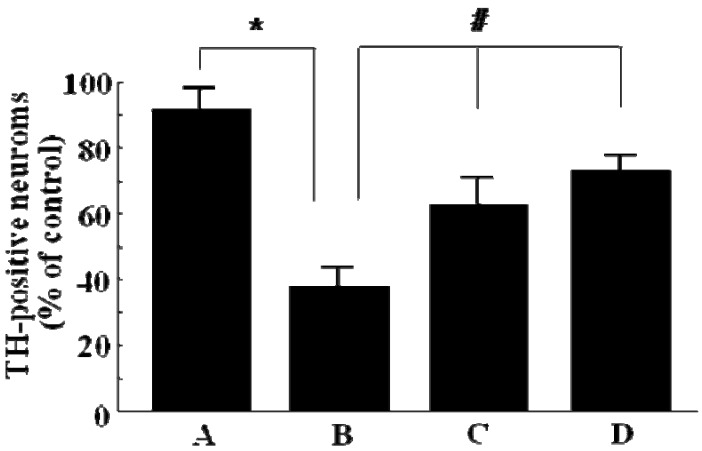
The number of surviving TH-immunopositive cells in the ipsilateral substantia nigra analyzed as a percentage of that in the intact contralateral side. GP-EX was administered to 6-OHDA-lesioned rats from day 3 for 28 days. Sham-lesioned groups (A), 6-OHDA-lesioned groups (B), GP-EX-administered groups [10 mg/kg (C) and 30 mg/kg (D)] in 6-OHDA-lesioned rats. TH-immunopositive cells were counted at 28 days after the injection of 6-OHDA and the cell survival was expressed as a percentage of ipsilateral versus contralateral substantia nigra (means ± S.E.M, four experiments). * *p* < 0.05 compared with sham groups; # *p* < 0.05 compared with 6-OHDA-lesioned groups (ANOVA followed by Tukey’s test).

The number of TH-immunopositive neurons in the ipsilateral side was analyzed as a percentage of that in the intact contralateral side. 6-OHDA lesioning caused a marked decrease in the number of TH-immunopositive neurons (40.2%) compared to the sham-lesioned control groups (Figure 2A and B). However, after 28 days of GP-EX treatment at 10 mg/kg and 30 mg/kg, the percentage of surviving TH-immunopositive neurons was 67.4% and 75.8%, respectively, compared to control groups (Figure 2C and D): GP-EX administration protected the loss of the number of TH-immunopositive neurons by 6-OHDA lesioning compared to the 6-OHDA-lesioned rats. No significant difference was observed in the groups administered GP-EX as compared to sham groups (data not shown).

### 2.2. The levels of dopamine, DOPAC, HVA and norepinephrine in striatum of 6-OHDA-lesioned rats by GP-EX administration

**Figure 3:**
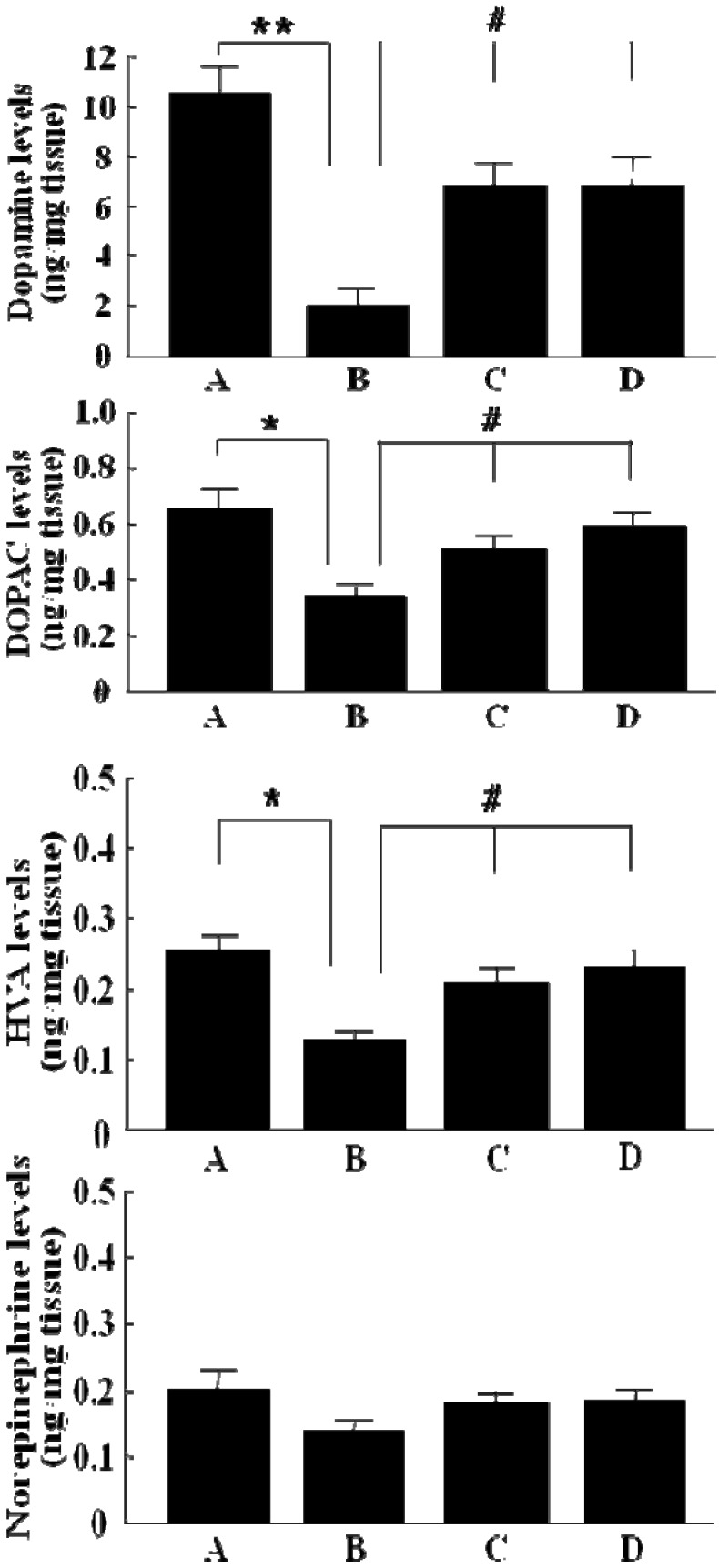
Effects of GP-EX on the levels of dopamine, DOPAC, HVA and norepinephrine in the striatum of 6-OHDA-lesioned rats. GP-EX (10 and 30 mg/kg) was administered orally to 6-OHDA-lesioned rats from day 3 post-lesion for 28 days. The levels of dopamine, DOPAC, HVA and norepinephrine in the striatum were determined by HPLC method. Results are represented as means ± S.E.M of four experiments. Sham-lesioned groups (A), 6-OHDA-lesioned groups (B), GP-EX-administered groups [10 mg/kg (C) and 30 mg/kg (D)] in 6-OHDA-lesioned rats. * *p* < 0.05 compared with sham groups; # *p* < 0.05 compared with 6-OHDA-lesioned groups (ANOVA followed by Tukey’s test).

As shown in Figure 3, a significant decrease in the levels of dopamine (19.1%, p < 0.01), DOPAC (52.3%, p < 0.05), HVA (47.1%, p < 0.05) and norepinephrine (67.4%) was observed in striatal regions of 6-OHDA-lesioned rats as compared to control groups. However, GP-EX administration (10 mg/kg) from day 3 post-lesion for 28 days to 6-OHDA-lesioned rats resulted in an improvement in the levels of dopamine (64.1%), DOPAC (77.9%), HVA (82.6%) and norepinephrine (88.1%) in lesioned rats as compared to control groups. Those treated with GP-EX (30 mg/kg) also showed a marked recovery activity of the levels of dopamine (65.0%), DOPAC (89.7%), HVA (90.2%) and norepinephrine (89.2%) as compared to control groups. The significant recovery activity of dopamine, DOPAC, HVA and norepinephrine levels by GP-EX administration (10 and 30 mg/kg) could be observed as compared to the 6-OHDA-lesioned groups (Figure 3). In addition, the levels of dopamine, DOPAC, HVA and norepinephrine in GP-EX-administered normal rats (those without 6-OHDA lesions) in striatum were not altered as compared to sham groups (data not shown). No differences were also seen on contralateral sides among sham groups, 6-OHDA-lesioned groups and 6-OHDA-lesioned groups administered with GP-EX (10 and 30 mg/kg, 28 days).

## 3. Discussion

Partial dopamine depletion by 6-OHDA in the striatum has been shown to be a good model for examining the effects of neurotrophic and neuroprotective therapies in the early and moderate stages of PD [17]. Among the various bioactive functions of *G. pentaphyllum,* it has been known to have antioxidant, anti-inflammatory, immune stimulant and anti-stress activities [11,12]. In this study, therefore, the neuroprotective functions of GP-EX, the ethanol extracts from the leaves of *G. pentaphyllum*, were investigated in 6-OHDA-lesioned rat models of PD. 

The symptoms of PD by 6-OHDA-administered rats for 14–28 days were confirmed by contralateral rotations after treatment with apomorphine, a dopamine receptor agonist, in the 6-OHDA-lesioned rats. Rats injected with saline solution did not display apomorphine-induced rotational movements. Upon confirming a rat model for PD, 6-OHDA-lesioned rats were compared to 6-OHDA-lesioned rats treated with GP-EX for 28 days starting at day 3 after 6-OHDA lesioning and the quantities of TH-related immunoreactive survival cells and the levels of dopamine, DOPAC, HVA and norepinephrine in the substantia nigra and striatum were investigated.

TH-immunopositive neuronal cell death by 6-OHDA lesions in the left substantia nigra was ameliorated by co-administration with GP-EX at 10 and 30 mg/kg (p.o.) for 28 days (Figure 1). The loss of the number of TH-immunopositive neurons in the ipsilateral side after 6-OHDA lesioning was recovered by GP-EX treatment compared to the 6-OHDA-lesioned groups (Figure 2). These results indicated that an oral administration of GP-EX exhibited a protective activity against the 6-OHDA-induced TH neuronal cell death.

In addition, the levels of dopamine, DOPAC, HVA and norepinephrine were decreased in striatal regions of 6-OHDA-lesioned rats. However, GP-EX administration (10 and 30 mg/kg) for 28 days to 6-OHDA-lesioned rats resulted in an improvement in the levels of dopamine, DOPAC, HVA and norepinephrine as compared to control groups (Figure 3), indicating that GP-EX administration protected against the reduction of dopamine, DOPAC, HVA and norepinephrine levels induced by 6-OHDA.

The 6-OHDA stereotaxic injection causes a progressive and dose-dependent dopaminergic cell death by the formation of various ROS in the ipsilateral side when compared to the contralateral control side [6]. In this study, no differences were seen on contralateral sides among sham groups, 6-OHDA-lesioned groups and 6-OHDA-lesioned groups administered with GP-EX (10 and 30 mg/kg, 28 days). Rats were administered GP-EX (10 and 30 mg/kg, p.o.) for 28 days after 6-OHDA lesions. The doses of GP-EX were determined by preliminary studies which showed that 10-50 mg/kg GP-EX exhibited a protective effect on transient cerebral ischemia in gerbil mice (data not shown). In addition, oral administration of GP-EX at doses up to 500 mg/kg/day for 28 days did not show toxic effects such as weight loss and death in rats [12]. It has also been reported that the water extract (750 mg/kg) of *G. pentaphyllum* does not produce any significant toxic effects in rats during a 6-month period of treatment [18]. 

GP-EX has been shown to have anti-stress functions by the improvement of body weight loss and the reduction of grip strength induced by electric footshock for three weeks in mice [12]. GP-EX also shows an immunomodulatory activity by preventing dexamethasone-induced immunosuppression and by increasing antitumor host defense when implanted into sarcoma tumor cells in mice [13]. These results may suggest reasons for the neuroprotective effects of GP-EX in 6-OHDA-lesioned rats. 

In our recent studies, the butanol fractions of GP-EX were found to be active for the protective effects on 6-OHDA-lesioned neurotoxicity in PD models. The bioactive components were also isolated from the butanol fractions of GP-EX and identified as gypenoside derivatives, including gynosaponin TN-1, gynosaponin TN-2, gypenoside XLV and gypenoside LXXIV [11,14,15,16]. The main components of GP-EX are also identified as gypenoside derivatives, which have been found to have an anti-inflammatory activity [19] and protective effects on hydrogen peroxide-induced oxidative stress [20,21] and glutamate-induced neurotoxicity [22]. We therefore propose that the gypenoside derivatives in GP-EX may play a role in the neuroprotective functions against a rat model of PD and will need to be studied further. 

Several studies have reported that 6-OHDA is detected in both human brain and rat [23,24] and its presence is due to the high levels of dopamine, hydrogen peroxide and free iron in dopaminergic neurons [25]. A non-enzymatic reaction between these elements may lead to the endogenous formation of 6-OHDA [26]. L-DOPA administration alone and in combination with Fe^2+^ also enhances 6-OHDA generation in rat brain [10]. Beside 6-OHDA, L-DOPA and dopamine can cause neurotoxicity by mediating oxidative stress in PC12 cells [27,28]. It is, therefore, proposed that antioxidants are a key to the prevention and control of the symptoms of PD [8,10,29]. Bioactive agents that enhance the bioavailability of dopamine or prevent its breakdown can provide protection against PD in humans and in animal models [30]. Considering our results, GP-EX in humans may be helpful in preventing the L-DOPA-induced adverse or toxic effects for PD, as well as slow down the progression of PD symptoms.

## 4. Experimental

### 4.1. Materials

6-OHDA, apomorphine hydrochloride, dopamine, DOPAC, HVA, norepinephrine, EDTA and L-ascorbic acid were purchased form Sigma Chemical Co. (St. Louis, MO, USA). TH antibody was obtained from Chemicon International (Temecula, CA, USA). Anti-mouse IgG and vectastain DAB and ABC kits were purchased form Vector Laboratories (Burlingame, CA, USA). All other chemicals were of analytical grade.

### 4.2. Preparation and treatment of GP-EX

*G. pentaphyllum* was obtained from Geochang (Gyungnam, Korea) and a voucher specimen of the herbal leaves of *G. pentaphyllum* was deposited at the herbarium of the College of Pharmacy, Chungbuk National University (Cheongju, Korea). The air-dried leaves of *G. pentaphyllum* (10 kg) were extracted with ethanol (70%, v/v) and the ethanol extracts were evaporated to dryness under reduced pressure and temperature (GP-EX, 1.05 g; yield, 10.5%, w/w) [12]. The dry GP-EX was suspended in water for the experiments. GP-EX (10 and 30 mg/kg) was administered to 6-OHDA-lesioned rats orally (p.o.) once a day, starting on day 3 post-lesion, for 28 days. Experimental rats were housed three to four per cage and had free access to food and water. 

### 4.3. Unilateral 6-OHDA lesion

Rats (Sprague-Dawley, male, 200–250 g) were purchased from Samtako Co. (Animal Breeding center, Osan, Korea). The experiments were performed according to the guidelines of the Care and Use of Laboratory Animals from the US Health and Human Services Department. Striatal 6-OHDA lesions were performed as previously described [31]. In brief, rats were anaesthetized intraperitoneally (i.p.) with Zoletil 50 (100 mg/kg, Virbac, Carros, France) and fixed to a stereotaxic apparatus (David Kopf Instruments, Tujunga, CA, USA) with an injection needle attached to a microsyringe (Hamilton 1701RN, USA) and an infusion pump (KDS310, KD scientific, Holliston, USA). The intranigral injection coordinates (−5.3 mm anterior–posterior, 1.9 mm left lateral, 7.8 mm ventral from the bregma) were taken from a rat brain atlas [32]. At a rate of 1 μL/min, 2 μl of 6-OHDA (8 μg/2 μL in 0.1% ascorbic acid-saline) was injected. The rats were divided into four groups and treated with GP-EX (10 and 30 mg/kg) or vehicle (0.9% NaCl) daily for four weeks after the 6-OHDA lesion. To confirm the formation of PD models, the rotational behavior of all rats induced by subcutaneous apomorphine administration (0.5 mg/kg) was tested at 2 weeks after 6-OHDA lesion and the number of contralateral rotation turns in 60 min was recorded [33].

### 4.4. TH immunohistochemistry

TH immunohistochemistry was performed according to the method of Ren and Sagar (1992) [17]. Rats were sacrificed 4 weeks after 6-OHDA lesioning and then perfused intracardially with 4% paraformaldehyde. After postfixation overnight, a Vibratome (Leica Microsystems, Wetzelar, Germany) was used to make 35μm-thick coronal brain sections. The tissue sections were incubated overnight in 5% normal goat serum, 0.1% Triton X-100 and mouse anti-TH (1:200; Chemicon). A1:250 dilution of biotinylated anti-rabbit IgG was used as a secondary antibody and then incubated with streptavidin-biotin-horseradish peroxidase complex. TH immunoreactivity was visualized using a DAB kit. Photomicrographs of TH and digitized bright-field images were captured using a Zeiss Axiophot microscope (Jena, Germany) (100× magnification). Cell counting was done using a computerized image analysis system. Analysis values obtained in the ipsilateral side were expressed as a percentage of those on the intact contralateral side. 

### 4.5. Determination of dopamine, DOPAC, HVA and norepinephrine

Determination of dopamine, DOPAC, HVA and norepinephrine levels was performed by the method of Tariq *et al.* [34]. The striatum (20%, w/v) was sonicated in 0.1 N perchloric acid containing EDTA (100 mM) and isoproterenol (1 × 10^−5 ^M) and then centrifugated at 15,000× g for 10 min at 4 °C. The supernatent was passed through a membrane filter (0.45 µm, Millipore, Waters) and the filtrate (100 μL) was injected into an HPLC system (Waters, Milford, MA, USA). 

### 4.6. Separation of gypenosides from GP-EX

The separation methods used to obtain the bioactive gypenosides from GP-EX included partition with water and butanol (1:1, v/v), Dianion HP-20 column chromatography, silica gel column chromatography, RP-18 column chromatography and subsequently prep-HPLC, [14,15,16]. Finally, four gypenosides (gynosaponin TN-1, gynosaponin TN-2, gypenoside XLV, gypenoside LXXIV) were isolated and identified by comparison with the spectral data (^1^H, ^13^C-NMR and ESI-MS spectra), physical constant and literatures, respectively [11,14,15,16].

### 4.7. Statistical analysis

All data were analyzed by one-way ANOVA followed by a Tukey’s test. Results are expressed as means ± S.E.M. in four experiments.

## 5. Conclusions

GP-EX displayed protective effects against neurotoxicity by reducing TH neuronal cell death and normalizing dopamine levels in 6-OHDA-lesioned rat model of PD. The physiological mechanisms using the bioactive components of GP-EX need to be examined in further studies.
